# Clinical and ultrasound features of a cohort of psoriasis patients without musculoskeletal symptoms: a prospective and multicentre study

**DOI:** 10.1093/rheumatology/keaf307

**Published:** 2025-06-04

**Authors:** Ana Belén Azuaga, Andrea Cuervo, Delia Reina, Paula Estrada-Alarcón, Lourdes Mateo, María Aparicio, Mireia Moreno, Marta Arévalo, Ana Láiz, Patricia Moya, Lucía Alascio, Josep Riera, José U Scher, Juan D Cañete, Julio Ramírez

**Affiliations:** Rheumatology Department, Hospital Clinic, Barcelona, Spain; Facultat de Medicina i Ciències de la Salut, Universitat de Barcelona (UB), Barcelona, Spain; Rheumatology Department, Hospital General de Granollers, Granollers, Spain; Rheumatology Department, Hospital Moisès Broggi, Barcelona, Spain; Rheumatology Department, Hospital Moisès Broggi, Barcelona, Spain; Rheumatology Department, Hospital Germans Trias i Pujol, Barcelona, Spain; Rheumatology Department, Hospital Germans Trias i Pujol, Barcelona, Spain; Rheumatology Department, Hospital Parc Taulí, Sabadell, Spain; Rheumatology Department, Hospital Parc Taulí, Sabadell, Spain; Functional Unit of Systemic Autoimmune Diseases (UFMAS), Rheumatology Department, Hospital de la Santa Creu i Sant Pau, Biomedical Research Institute (IIB Sant Pau), Universitat Autònoma de Barcelona, Barcelona, Spain; Functional Unit of Systemic Autoimmune Diseases (UFMAS), Rheumatology Department, Hospital de la Santa Creu i Sant Pau, Biomedical Research Institute (IIB Sant Pau), Universitat Autònoma de Barcelona, Barcelona, Spain; Rheumatology Department, Hospital Clinic, Barcelona, Spain; Dermatology Department, Hospital Clinic, Barcelona, Spain; Department of Medicine, Division of Rheumatology, NYU Grossman School of Medicine, New York, NY, USA; Rheumatology Department, Hospital Clinic, Barcelona, Spain; Fundació Clinic de Recerca Biomèdica and IDIBAPS, Barcelona, Spain; Rheumatology Department, Hospital Clinic, Barcelona, Spain

**Keywords:** psoriatic arthritis, psoriasis, ultrasound, early diagnosis

## Abstract

**Objectives:**

To evaluate clinical and ultrasound (US) features related to psoriatic arthritis (PsA) development in psoriasis (PsO), patients without musculoskeletal (MSK) symptoms and no systemic treatment.

**Methods:**

Prospective study including PsO patients followed by dermatologists. Clinical and US data were collected at baseline and during follow-up by rheumatologists.

**Results:**

Seventy-eight patients with PsO were included. Mean disease duration was 15.1 years (SD ± 13.4); most had mild PsO (82%), onychopathy was present in 36 (39.7%) and overweight/obesity in 38 patients (48.7%). In the US evaluation, nine patients (11.5%) had Power Doppler grade 1 at joints, 56.4% had calcifications and 30.4% bursitis at enthesis. Sixty patients completed the study. After a median of 76.60 months (IQR 39.34–85.25), 34 patients (56.6%) developed MSK symptoms. They had higher BMI (*P* = 0.013), abdominal circumference (*P* = 0.022), scored higher for pain (*P* = 0.047) and fatigue (*P* = 0.011). Their baseline US showed a higher total US score (*P* = 0.037). Five patients (8.3%) developed MSK inflammatory symptoms, and four met CASPAR criteria (5.5%). The mean time from baseline to PsA diagnosis was 20.20 months (SD ± 12.02). US bursitis was present in 80% of patients developing inflammatory symptoms (*P* = 0.049).

**Conclusions:**

In a cohort of patients with mild PsO, systemic therapy-naive and no MSK symptoms, incidence of PsA was 1% per year. US bursitis at enthesis was related to the development of symptoms suggestive of PsA. Patients who developed MSK symptoms had higher BMI, fatigue and pain VAS scores at baseline and could constitute a subgroup with higher risk for transition to PsA.

Rheumatology key messages

- Incidence of PsA among patients with mild PsO without MSK symptoms is low.
- Ultrasound bursitis at enthesis could be the first manifestation of PsA.
- Patients who developed MSK symptoms had higher BMI, fatigue and pain VAS scores, and more abnormal US findings.

## Introduction

Psoriasis (PsO) and psoriatic arthritis (PsA) are chronic immune-mediated inflammatory diseases that share genetic susceptibility, pathogenic pathways and therapeutic targets. PsO affects the skin, has a prevalence of about 3% [1, 2] and usually precedes PsA by 5–10 years in most patients [3–5]. PsA is a heterogeneous disease that affects not only the skin but also multiple musculoskeletal (MSK) domains, including peripheral joints, axial involvement, entheses and dactylitis [6]. Uveitis and inflammatory bowel diseases (Crohn’s and ulcerative colitis) are also associated to PsA. To capture all this complexity, the concept of psoriatic disease has been implemented. The prevalence of arthritis in PsO patients varies across studies, with estimates ranging from 8% to 30% [2, 7, 8]. Moreover, it has been reported that ∼20% of patients with PsO have undiagnosed PsA [9, 10], and up to 47% have MSK symptoms [11, 12].

In addition to genetic predisposition, certain clinical characteristics, such as obesity and specific phenotypes of PsO (including onychopathy, scalp involvement and PsO extension), have been associated with higher risk of development of PsA [5, 13–15]. To facilitate rapid referral and early diagnosis in this population, dermatologists and rheumatologists have developed and validated several questionnaires to screen for PsA in patients with PsO [16–19].

Although the progression of PsA may vary among patients, it often follows a progressive course that significantly impacts their lives [20]. Continuous inflammation promotes the development of bone erosions, leading to joint destruction, disability and a worsening of quality of life. To prevent these outcomes, early diagnosis and treatment are crucial [21]. Despite advances in technology, diagnosing PsA among patients with PsO remains challenging and frequently the diagnosis of PsA is delayed, which could take up to 5 years after the development of initial MSK symptoms [22, 23].

The current diagnostic approach relies primarily on clinical findings and laboratory tests, particularly erythrocyte sedimentation rate (ESR) and C-reactive protein (CRP), which are non-specific and can be normal in some patients [24–26]. Ultrasound (US) of joints and entheses is a rapid and non-invasive tool for the diagnosis and monitoring of rheumatological diseases, as it identifies inflammatory lesions and structural damage. In addition, US can detect subclinical synovitis and enthesitis in PsO patients without MSK symptoms [27–29], as well as in patients with arthralgia [30].

At present, prediction of the transition from PsO to PsA remains an unmet medical need. Moreover, most of the existing studies focusing on this topic are retrospective. In our prospective observational study, we aimed to assess potential MSK symptoms and objective signs of inflammation to establish risk factors for the development of PsA, using clinical and US assessments in a cohort of patients with mild psoriasis, without MSK symptoms and off systemic therapy at inclusion.

## Methods

### Patients

We included patients with PsO attending to the Dermatology Department of five academic hospitals in Barcelona, Spain. Patients with mild, moderate or severe PsO who were not receiving systemic treatment at the time of inclusion were included. Patients with PsO at any location (including scalp or nail) as well as any type (guttate, pustular, inverse, erythrodermic) were included.

A comprehensive anamnesis and a medical history review were conducted to exclude patients who currently or previously had MSK symptoms, although the visual analogue scale (VAS) for pain was not used as an exclusion criterion.

### Study design

This is a prospective study including patients with PsO evaluated by rheumatologists from October 2015 to June 2024. The study consisted of two phases: the baseline visit, and a second follow-up was scheduled after 5 years. However, some patients had an early end to their study appointment in order to collect the necessary data for this analysis or due to the development of MSK symptoms over time, for which they consulted directly with the rheumatologist. In both phases, data were collected and evaluated to determine whether they met the Classification of Psoriatic Arthritis (CASPAR) criteria. This study was in accordance with the principles of the Declaration of Helsinki and approved by the Ethics Committee of the Hospital Clinic of Barcelona (HCB/2015/0336). Participants provided informed consent.

### Data collection

The following was collected at baseline and during follow-up visits: family history (PsO, spondyloarthritis, rheumatoid arthritis and inflammatory bowel disease), year of onset, PsO type and severity, treatments received and current treatment; toxic habits (smoking, alcohol); anthropometric measurements [weight, height, abdominal circumference and body mass index (BMI)]; comorbidities (diabetes mellitus, glucose intolerance, hyperlipidemia, hypertension, ischemic heart disease, stroke, depression, inflammatory bowel disease). Patients reported questionnaires including the Psoriasis Epidemiology Screening Test (PEST) for suspected PsA; Bath Ankylosing Spondylitis Disease Activity Index (BASDAI) to evaluate axial involvement; Psoriatic Arthritis Impact of Disease (PsAID) for disease impact, and Physician’s Global Assessment (PGA). Clinical evaluation was made of tender and swollen joints counts of 68/66 (TJC and SJC, respectively), Leeds Enthesitis Index (LEI) and number of fingers with dactylitis. To evaluate skin domains activity, we used body surface area (BSA). Blood samples were collected to measure ESR, CRP and basic biochemistry, including metabolic profile.

### Ultrasound assessment

For each patient, a US evaluation was performed at baseline and at follow-up visit. All studies were performed by an expert rheumatologist in this technique (JRa). The examination included the following recesses of the following bilateral joints: elbows (anterior and posterior recess), wrists (dorsal radiocarpal and mediocarpal recess), metacarpophalangeal joints second to fifth (dorsal recess), knees (suprapatellar and external parapatellar recess), tibiotalar (anterior recess), and metatarsophalangeal joints second to fifth (dorsal recess) to complete a total of 40 joints. The following US findings were evaluated in each synovial recess with the following definitions: synovial hypertrophy (SH) and Power Doppler (PD) [31]. Regarding the previous variables, each of the US findings was assessed for: presence/absence and semiquantitative grade from 0 to 3 [32], adapted to each joint as appropriate. Strict US synovitis was defined as the presence of SH ≥ 2 + PD. Additionally, the US examination of the following bilateral entheses was performed: brachial triceps tendon, distal quadriceps tendon, proximal patellar tendon, distal patellar tendon, distal Achilles tendon and proximal plantar fascia to complete a total of 10 entheses. In each of them, the presence of thickening, echotexture, calcifications/enthesophytes, erosions, bursitis and PD signal were assessed. For the quantification of these findings, the Madrid Sonographic Enthesis Index (MASEI) [33] was used. For the US evaluations, an Esaote My Lab Twice equipment with a linear probe of 5–18 MHz was used.

### Statistical analysis

Patient demographics and disease characteristics were analysed using standard descriptive statistics. Categorical data are presented as percentages, while continuous variables were reported as mean and standard deviation (SD). There was greater variation among the outliers in evaluating the follow-up time, which led us to use the median [interquartile range (IQR)] as the representative value of central tendency. We used χ2 to compare categorical variables and the *t* test to compare quantitative and categorical variables. Outcomes of interest were PsA diagnosis (meeting CASPAR criteria) and new-onset MSK symptoms. The statistical analysis was performed using SPSS Statistics 27 (Chicago, IL, USA).

## Results

### Demographic and clinical characteristics of the PsO cohort at baseline

**Figure 1.**
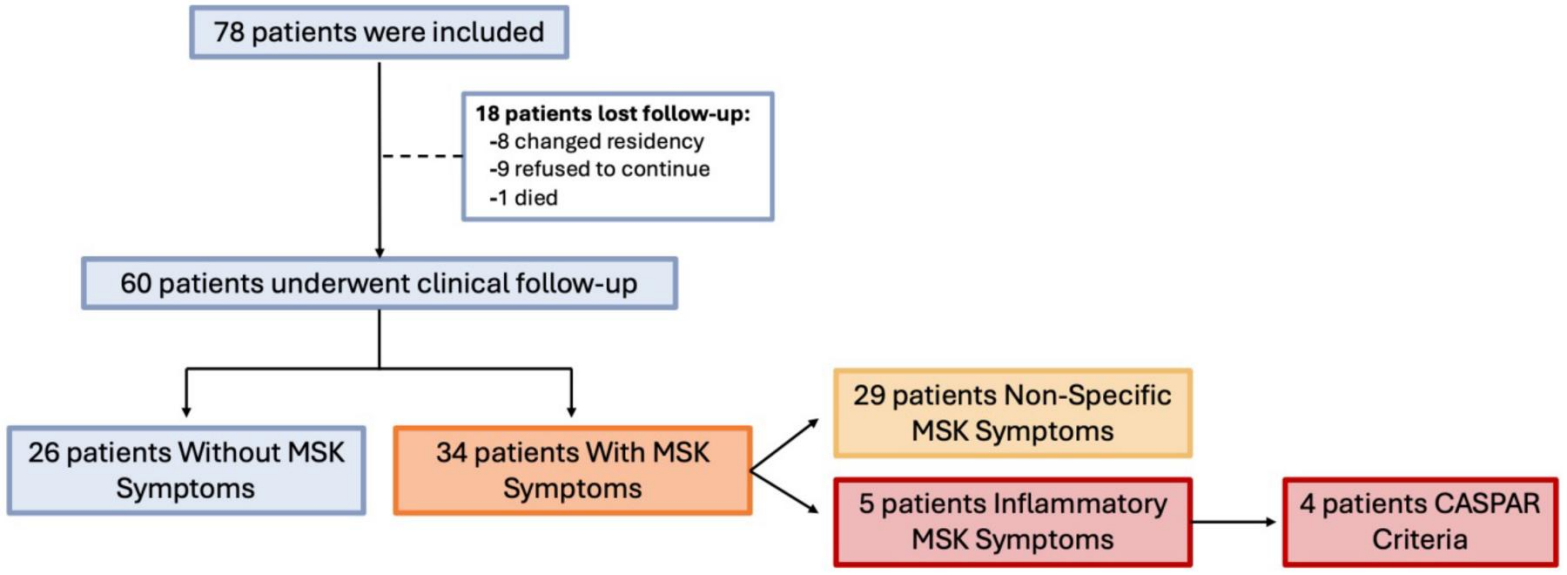
Patient inclusion and follow-up

A total of 78 patients with PsO were included. Eighteen patients did not have a follow-up visit due to geographic distance or personal decision due to the lack of MSK symptoms. Fig. 1 shows the flow diagram of study design, inclusion criteria and follow-up outcomes. None of the participants had MSK symptoms during anamnesis or were taking systemic therapy at baseline. Thirty-six patients (46.1%) used topical therapy and 20 patients (25.6%) were receiving phototherapy. Sixty-four patients (82%) had mild PsO, and most of them had plaques distributed over extensor surfaces. Scalp, inverse and palmoplantar PsO were present in 40 (51.2%), four (5.1%) and two (2.5%) patients, respectively. Onychopathy was present in 36 patients (39.7%). Thirty-eight (48.7%) patients were overweight/obese and had significantly more onychopathy (22% *vs* 14%, *P* = 0.016). Additional demographic and clinical characteristics are described in Table 1. Patients with onychopathy had significantly higher PEST scores [1.34 (0.68) *vs* 0.47 (0.72), *P* < 0.001]. In addition, PEST scores weakly correlated with more extensive skin involvement (BSA) (r = 0.354, *P* = 0.004) and greater BMI (r = 0.241, *P* = 0.038).

**Table 1.** Demographic and clinical characteristics

	Baseline	Follow-up	*P*
N	78	60	
Female, *n* (%)	46 (59.0)	33 (42.3)	**<0.001**
Age, mean (SD)	47.12 (13.98)	51.78 (13.92)	**<0.001**
Current smoker, *n* (%)	30 (38.5)	15 (19.2)	**0.001**
Overweight/Obese, *n* (%)	38 (48.7)	20 (25.6)	**<0.001**
BMI, mean (SD)	25.62 (4.90)	26.67 (4.94)	**<0.001**
Duration of PsO (years), mean (SD)	15.13 (13.45)	20.64 (14.08)	**<0.001**
BSA, mean (SD)	4.59 (5.93)	2.80 (4.55)	0.872
CRP, mean (SD)	0.24 (0.18)	0.55 (0.78)	0.107
ESR, mean (SD)	9.09 (5.30)	10.09 (6.34)	0.774
PsAID, mean (SD)	1.86 (1.94)	1.15 (1.52)	**0.029**
PEST, mean (SD)	0.91 (0.84)	0.71 (0.799)	0.121
BASDAI, mean (SD)	1.14 (1.32)	1.73 (2.03)	**0.024**

BASDAI: Bath Ankylosing Spondylitis Disease Activity Index; BMI: body mass index; BSA: body surface area; CRP: C-reactive protein; ESR: erythrocyte sedimentation rate; PEST: Psoriasis Epidemiology Screening Test; PsAID: Psoriatic Arthritis Impact of Disease; PsO: psoriasis; SJC: swollen joint count; SD: standard deviation. *P* values <0.05 are marked in bold.

### Ultrasound characteristics of the PsO cohort at baseline

Inflammatory changes were infrequent in the US evaluation. Nine patients (11.5%) showed PD signal (all grade 1) at joints, and two patients (6.4%) met strict synovitis criteria (SH ≥2 + PD). The mean MASEI at baseline was 5.90 (SD 5.62). Structural findings were more frequent at entheses: erosions were found in 16 patients (20.5%), increased thickness in 13 patients (16.6%), abnormal US structure in 18 patients (23%) and calcifications (predominantly at Achilles and quadriceps entheses) in 44 (56.4%) patients. Regarding inflammatory findings at entheses, seven (9%) patients had PD and 24 (30.4%) patients had distension of bursa without PD, mainly at Achilles and distal patellar ligament entheses.

### Clinical characteristics of the PsO cohort with follow-up

Follow-up data were available for 60 patients (76.9%). After a median follow-up period of 76.60 months (IQR 39.34–85.25), 19 (24.1%) patients had initiated systemic treatment due to their dermatological condition: 15 with biological therapy, three with methotrexate and two with acitretin. On the other hand, 39 were on topical therapy and one on phototherapy. The mean time from the baseline visit to the initiation of systemic treatment was 36.67 months (SD 29.20). Table 2 details the types of systemic treatments the patients were receiving at follow-up.

**Table 2.** Treatment changes and type at follow-up

N	60
Change in treatment, *n* (%)	24 (40)
Topical, *n* (%)	39 (65)
Phototherapy, *n* (%)	1 (1.6)
use of bDMARDs, *n* (%)	15 (25)
Systemic treatment, *n* (%)	20 (33.3)
MTX, *n* (%)	3 (5)
Acitretin, *n* (%)	2 (3.3)
TNFi, *n* (%)	3 (5)
IL23i, *n* (%)	4 (6.6)
IL17i, *n* (%)	8 (13.3)

bDMARDs: biological disease-modifying anti-rheumatic drugs; IL17i: interleukin 17 inhibitors; IL23i: interleukin 23 inhibitors; MTX: methotrexate; N: number of patients; TNFi: tumor necrosis factor inhibitors.

Patients needing systemic therapy during follow-up were predominantly overweight/obese (48% *vs* 21%, *P* = 0.05) and had significantly higher skin activity (BSA) at baseline (*P* =0.032). These patients also experienced a deeper life impact of the disease at baseline, as shown by their higher scores on several items included in the PsAID questionnaire (work and/or leisure activities, functional capacity, coping, embarrassment and/or shame, and social participation). Table 3 shows the differences between patients who required systemic treatment and those who did not.

**Table 3.** Differences in baseline PsAID VAS scores between patients requiring systemic treatment and those not requiring systemic treatment

​	Systemic DMARDs	Non-Systemic DMARDs	*P*
PsAID, mean (SD)	2.09 (1.99)	1.48 (1.18)	0.234
Pain, mean (SD)	1.66 (2.19)	1.63 (2.25)	0.916
Fatigue, mean (SD)	2.44 (3.39)	2.18 (2.63)	0.858
Skin problems, mean (SD)	3.55 (3.01)	3.18 (2.72)	0.702
Work and/or leisure activities, mean (SD)	2.05 (3.09)	0.57 (1.36)	0.057
Functional capacity, mean (SD)	1.38 (2.74)	0.47 (1.46)	0.160
Discomfort, mean (SD)	1.50 (2.77)	1.60 (2.49)	0.694
Sleep disturbance, mean (SD)	2.05 (3.09)	1.50 (2.81)	0.383
Coping, mean (SD)	2.66 (3.49)	0.78 (1.45)	**0.047**
Anxiety, fear and uncertainty, mean (SD)	2.00 (3.37)	1.13 (2.09)	0.634
Embarrassment and/or shame, mean (SD)	2.77 (3.71)	0.97 (1.80)	0.100
Social participation, mean (SD)	1.05 (2.60)	0.15 (0.67)	0.054
Depression, mean (SD)	0.27 (0.82)	0.31 (1.16)	0.736

PsAID: Psoriatic Arthritis Impact of Disease questionnaire; VAS: Visual Analogue Scale; DMARD: disease-modifying anti-rheumatic drug; SD: standard deviation. *P* values <0.05 are marked in bold.

On the other hand, two patients scored >3 on the PEST questionnaire and three patients had a MASEI score >18 at baseline; all of them developed MSK symptoms but none met the CASPAR criteria for PsA. However, baseline US findings in both joints and entheses were not associated with the extent of skin plaques (BSA), subjective assessments of pain or fatigue, acute phase reactants (CRP and ESR) or changes in treatment during follow-up.

### Development of musculoskeletal symptoms

**Figure 2.**
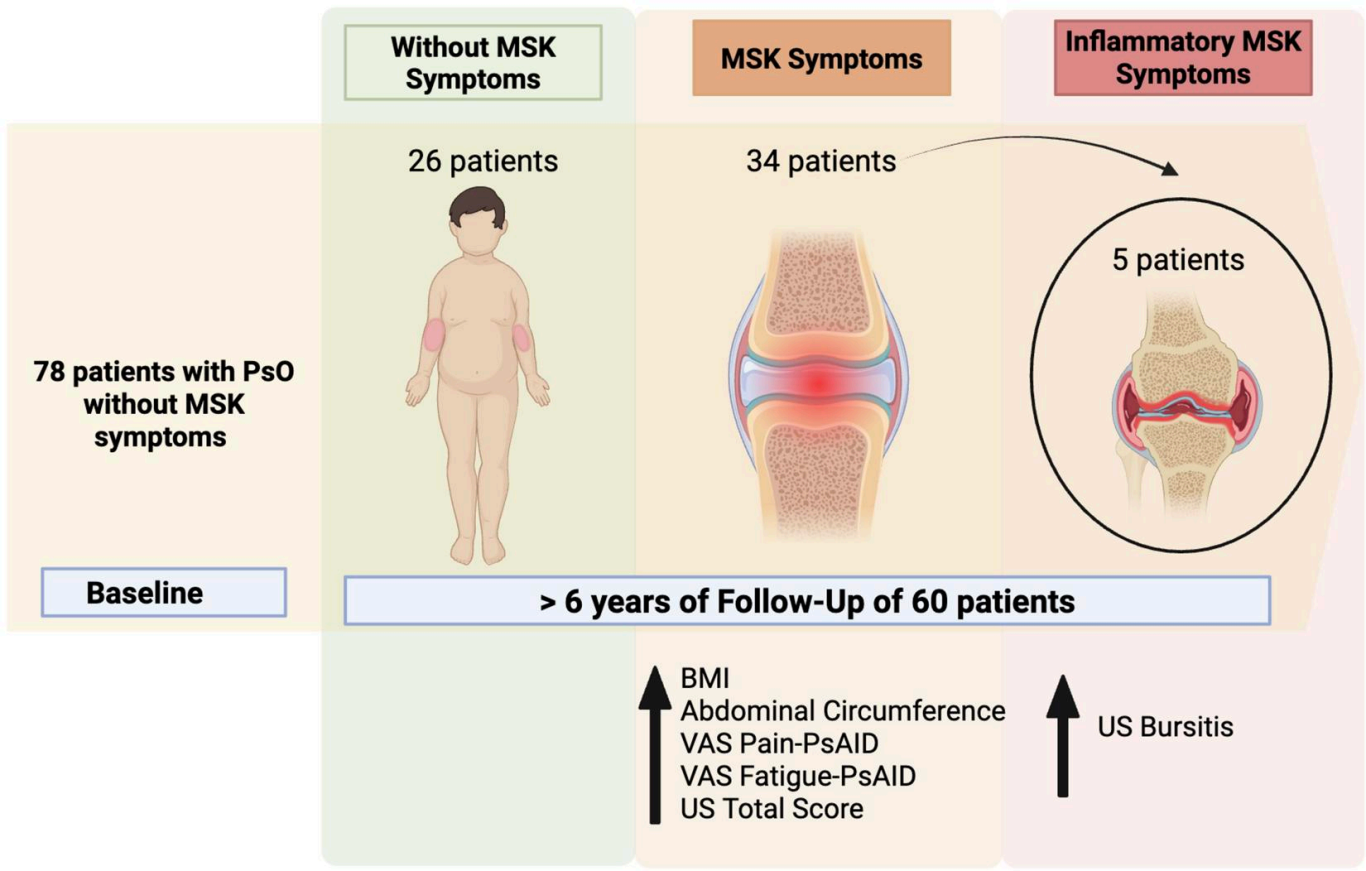
Evolution of MSK symptoms over the follow-up and baseline-associated characteristics in patients with PsO

Among the 60 patients who completed the study follow-up, five patients (8.3%) developed inflammatory MSK symptoms suggestive of PsA according to their rheumatologist. These patients were all men, with a mean age of 56.9 years (SD ± 10.33) at the inflammatory MSK symptoms onset and had a mean PsO duration of 7.56 years (SD±7.05). The mean duration from the baseline visit to the diagnosis of PsA was 20.20 months (SD±12.02) and none of these patients were on systemic therapy. Four out of 60 (6.6%) patients met the CASPAR criteria: three had oligoarticular pattern. Fig. 2 shows the evolution of MSK symptoms over the follow-up. Only one patient had a PEST score of 3 at baseline visit. Two of them received DMARDs, one received local glucocorticoids injections and the remaining patient experienced an isolated episode of dactylitis that was treated with local glucocorticoids injection. The fifth patient who reported inflammatory arthralgia (axial and peripheral) without evidence of arthritis is currently on demand NSAIDs. All five had scalp involvement, and one had inverse PsO. Three of them were obese, and one had onychopathy. Only one of these patients was a smoker. At baseline, distension of the bursa at enthesis was present in the US of four out of five (80%) patients (*P* = 0.049) developing inflammatory arthralgia suggestive of PsA. Three had distal patellar ligament bursitis, and one patient had sub-Achilles bursitis. No significant differences were found in other US items, although baseline US joint scores in these patients were slightly above the mean of the whole population.

Overall, 34 (56.6%) patients reported MSK symptoms during the follow-up (both inflammatory and non-inflammatory). At baseline, these patients had significantly higher BMI and abdominal circumference than patients not developing MSK symptoms (26.5 kg/m^2^  *vs* 23.5 kg/m^2^, *P* = 0.013 and 105.3 cm *vs* 97.2 cm, *P* = 0.022, respectively). In addition, they scored higher on pain [2.14 (2.52) *vs* 0.91 (1.56), *P* = 0.047] and fatigue [3.25 (3.22) *vs* 1.21 (2.21), *P* = 0.011] components of the baseline PsAID questionnaire. Neither onychopathy nor scalp PsO were related to the development of MSK symptoms. In addition, the US examination at baseline showed numerically higher scores for both joint SH and PD, with the total joint US score being significantly higher in these patients than in patients not developing MSK symptoms (*P* = 0.037). No significant differences were found between patients starting or not starting systemic therapy regarding the onset of MSK symptoms. Table 4 shows the differences between patients with MSK symptoms *vs* non-MSK symptoms.

**Table 4.** Clinical and ultrasound baseline characteristics in patients who developed MSK symptoms *vs* non MSK symptoms

	MSK symptoms	Non MSK symptoms	*P*
N	34	26	
Female, *n* (%)	22 (35.3)	12 (46.1)	0.193
Age, mean (SD)	47.41 (12.44)	43.90 (15.77)	0.340
Onychopathy, *n* (%)	16 (47.05)	17 (58.62)	0.601
BMI, mean (SD)	26.58 (5.11)	23.51 (4.15)	**0.016**
Pelvic waist length, mean (SD)	105 (11.61)	91.22 (11.90)	**0.022**
Duration of PsO (years), mean (SD)	15.02 (12.77)	14.92 (14.13)	0.978
BSA, mean (SD)	5.68 (7.80)	3.39 (3.20)	0.154
TJC, mean (SD)	0.24 (0.55)	0.23 (0.81)	0.980
SJC, mean (SD)	0.03 (0.17)	0.12(0.43)	0.294
CRP, mean (SD)	0.26 (0.18)	0.23 (0.21)	0.597
ERS, mean (SD)	8.68 (4.93)	8.71 (4.57)	0.989
PsAID, mean (SD)	1.85(1.67)	1.47 (1.15)	0.323
PEST, mean (SD)	1.06 (0.81)	0.73 (0.66)	0.100
BASDAI, mean (SD)	1.42 (1.33)	1.00 (1.18)	0.210
MASEI, mean (SD)	5.38 (6.60)	6.65 (5.28)	0.425
SH US, mean (SD)	3.41 (2.91)	2.00 (2.56)	0.055
PD US score, mean (SD)	0.41 (0.78)	0.15 (0.46)	0.118
US total score, mean (SD)	3.79 (3.23)	2.15 (2.70)	**0.037**

BASDAI: Bath Ankylosing Spondylitis Disease Activity Index; BMI: body mass index; BSA: body surface area; CRP: C-reactive protein; ERS: erythrocyte sedimentation rate; MASEI: Madrid Sonographic Enthesis Index; PD: power Doppler; PEST: Psoriasis Epidemiology Screening Test; PsAID: Psoriatic Arthritis Impact of Disease; SH: synovial hypertrophy; SJC: swollen joint count; TJC: tender joint count. P values <0.05 are marked in bold.

## Discussion

This prospective study aimed to analyse clinical and US findings related to the development of PsA in a cohort of PsO patients with mostly mild skin involvement. For that purpose, we collected data from patients without systemic treatment (avoiding treatment bias) and without MSK symptoms (excluding mechanical problems, fibromyalgia or diffuse pain mimicking prodromal symptoms of PsA). To our knowledge, this is the first study to evaluate all the items in the PsAID in patients with PsO, which allows the measurement of several clinical domains informing about the impact of the psoriatic disease on the patient.

This prospective study, with a median follow-up of >6 years, found only 6.6% of patients meeting CASPAR criteria for PsA (1% per year). This incidence is much lower compared with two previous studies, in which 30% and 21% of patients with PsO developed PsA after 12 months and a median of 28 months of follow-up, respectively. However, although these two studies included patients with PsO without evidence of PsA, MSK symptoms were not an exclusion criterion. As expected, pain was one of the main factors associated with the development of PsA after a short follow-up period [10, 34]. Another larger study including 464 patients found an incidence of PsA of 2.7% per year [35]. However, in contrast to our cohort, a subgroup of the PsO patients were referred to the rheumatologist to rule out any MSK inflammatory involvement. Although the rheumatologist found no clinical inflammatory signs, US confirmation was not performed. In addition, 13.2% of patients had moderate-severe PsO. They found that PsO severity, nail pitting and systemic retinoid medication – all surrogate markers of disease severity – were predictors of PsA development [35].

Recently, another study found that among patients with subclinical PsA – defined as inflammatory arthralgia not explained by other diagnoses – the probability of new-onset PsA was 9.4% at month 12 and 22.7% at month 36 [36]. In our study we excluded PsO patients with arthralgia to minimize the risk of including PsO patients in a prodromal phase of PsA. On the other hand, we only included patients without systemic therapies. DMARDs, especially biological therapy, have been proposed in a few retrospective observational studies as a protective factor for PsA development [37, 38] whereas other retrospective study found that bDMARDs were linked to the development of PsA [39]. These authors suggested an indication bias, meaning that PsO patients starting bDMARDs were in fact at a high risk of developing PsA, or even that PsA was already present at inclusion but still not diagnosed [39]. However, exclusion of patients undergoing systemic therapies could led to the inclusion of predominantly mild PsO cases, which could reduce the risk of developing PsA, as skin severity is a known risk factor for PsA [5, 13–15]. Nevertheless, within our cohort there were patients with other risk factors associated with PsA development, as obese patients that had higher PEST scores and more onychopathy at baseline.

Overall, the inclusion of patients without MSK symptoms and no systemic therapy likely explains the low incidence of PsA in our cohort, despite the long follow-up period. Although only four patients met the CASPAR criteria for PsA, more than half of the cohort developed MSK symptoms during follow-up, including both inflammatory and non-inflammatory symptoms. These patients had higher BMI and scored significantly higher for pain and fatigue in the PsAID at baseline. Pain and fatigue have already been considered risk factors for developing PsA. Eder *et al.* in a longitudinal study of PsO patients, observed that the presence of arthralgia, heel pain, fatigue, and stiffness are early symptoms associated with the subsequent development of PsA [40]. However, in our cohort, it was more strongly associated with non-specific MSK symptoms rather than PsA, possibly due to the low incidence of PsA in our study population.

Finally, we analysed US findings as predictors of PsA, since subclinical imaging findings in both entheses and joints have already been related to the future development of PsA [27, 41–43]. Accordingly, patients developing PsA or inflammatory arthralgia in our cohort showed higher US scores at baseline, although the low number of patients reaching these outcomes limited the statistical analysis. While inflammatory US findings were not strongly linked to PsA in our cohort, bursitis at the entheses was significantly associated with the development of inflammatory MSK symptoms. After expanding the population to all patients developing MSK symptoms, significant differences were found in baseline global US joint scores (including both SH and PD). US abnormalities in our cohort were relatively common, although most of them were structural changes, mainly calcifications in the entheses, particularly in the lower extremities, which may be attributed not only to PsA but also to mechanical overload. As expected, patients with a higher BMI exhibited more advanced US findings, with higher MASEI and joint US scores. More than half of the patients with PsO had calcifications, and about 20% had other structural findings, such as increased thickness or abnormalities in the structure of the enthesis. These results should be interpreted with caution due to the limited cohort size. On the other hand, we found that US findings were more often associated with non-inflammatory MSK symptoms, which limits its usefulness in identifying PsA in PsO patients without inflammatory MSK symptoms.

Despite the presence of inflammatory findings such as US PD grade 1 (11%) and two cases meeting criteria for subclinical synovitis (SH ≥ 2+PD), no association was found with the occurrence of MSK symptoms during follow-up. Only grey-scale bursa distension at enthesis (present in one third of the cohort) was significantly associated with the occurrence of inflammatory symptoms, which reinforces the idea of enthesis as a major area of interest in the occurrence of PsA [44, 45]. However, the small number of patients included in this study recommends a cautious interpretation of this finding.

The limitations of our study are mainly determined by the sample size, which is relatively small, potentially underestimating the incidence of PsA in patients with PsO. Additionally, the exclusion of patients with MSK symptoms and systemic therapy probably excluded those with a higher risk of developing PsA. Throughout the study, a significant proportion of patients initiated DMARD therapy by their dermatologists, including biologics, which could have negatively impacted the development of PsA symptoms, although multivariable analysis did not find it to be a significant factor related to new-onset symptoms (data not shown).

Conversely, to our knowledge, this is the longest prospective follow-up study conducted in a cohort of PsO patients with clinical, US and PsAID to date. Long follow-ups are challenging in patients without MSK clinical symptoms, considering that many patients with PsO and no MSK symptoms are reluctant to adhere to unnecessary long follow-up visits with rheumatologists. Moreover, our strict inclusion criteria may have excluded patients who were already in transition to PsA. Future research should include more diverse patients and apply multi-omic technologies to detect cellular and/or molecular biomarkers to elucidate the biological mechanisms defining the transition from PsO to PsA.

## Conclusion

Around half of the patients with PsO developed MSK symptoms during the follow-up and they had significantly higher BMI, more joint US findings and scored higher in pain and fatigue scales at baseline PsAID questionnaire, defining a subgroup with potentially higher risk to develop PsA. On the other hand, US bursitis at enthesis was related to the onset of MSK symptoms suggestive of PsA. Our results indicate that in PsO, patients without arthralgia and non-specific US findings, the likelihood of developing PsA over time is very low. Predicting PsA remains challenging, highlighting the need for continued research, with prospective clinical studies and the development of more precise biomarkers of transition to PsA.

## Data Availability

All data are available from the corresponding author upon reasonable request.
